# Impact of kimura-takemoto atrophy classification on first-line *H. pylori* eradication: a retrospective cohort study

**DOI:** 10.3389/fmed.2026.1864653

**Published:** 2026-06-19

**Authors:** Dongchu Wang, Xiangwu Ding, Aixiang Wang

**Affiliations:** Department of Gastroenterology, Wuhan Fourth Hospital, Wuhan, China

**Keywords:** clarithromycin, eradication therapy, gastric atrophy, *helicobacter pylori*, kimura-takemoto classification

## Abstract

**Objective:**

We sought to determine whether the endoscopic grade of gastric atrophy (according to Kimura-Takemoto) affects the likelihood of successful first-line *H. pylori* eradication.

**Methods:**

We conducted a retrospective analysis of consecutive patients hospitalized at Wuhan Fourth Hospital between November 2021 and November 2025. Eligible individuals had a positive urea breath test confirming *H. pylori* infection, were diagnosed with chronic atrophic gastritis via gastroscopy, received a bismuth-containing quadruple regimen as first-line therapy, and returned for a follow-up breath test at least 4 weeks post-treatment. Prior to therapy (within 28 days), each patient underwent high-definition white-light gastroscopy. Two independent endoscopists, unaware of patient allocation, retrospectively reviewed all images to grade atrophy using the Kimura-Takemoto system. Patients were assigned to either the closed-type (C-type) or open-type (O-type) atrophy group. The primary endpoint was eradication failure, defined as a positive follow-up breath test. Logistic regression (univariate and multivariate) was used to identify factors independently linked to treatment failure.

**Results:**

A total of 154 patients completed follow-up and were included. The overall eradication rate was 76.6% (118/154). In the open-type atrophy group (*n* = 26), the eradication rate was only 42.3% (11/26), significantly lower than the 83.6% (107/128) observed in the closed-type group (*P* < 0.001). After adjusting for confounders such as body weight and age, multivariate analysis revealed that endoscopic open-type atrophy was independently associated with eradication failure (OR = 8.287, 95% CI: 3.150–21.804, *P* < 0.001).

**Conclusion:**

The extent of endoscopic gastric mucosal atrophy independently predicts a lower efficacy of first-line *H. pylori* eradication. For patients with Kimura-Takemoto open-type atrophy, clarithromycin-containing quadruple regimens should be used cautiously. Alternative clarithromycin-free regimens (e.g., tetracycline-, metronidazole-, or levofloxacin-based therapies, or high-dose amoxicillin with PPI/vonoprazan) or susceptibility-guided individualized therapy are recommended in clinical practice.

## 1 Introduction

*Helicobacter pylori* infection is a globally common chronic bacterial condition and represents the most significant modifiable risk factor for chronic active gastritis, peptic ulcer disease, gastric mucosa-associated lymphoid tissue lymphoma, and gastric cancer (1–4). Therefore, successful eradication is essential to halt the progression of these diseases. However, eradication success is influenced by a complex interplay of bacterial factors (e.g., primary antibiotic resistance) and host characteristics (5–7). Among host factors, the degree of gastric mucosal atrophy has increasingly been recognized as a key determinant of treatment outcomes (8, 9). For example, Kamada et al. (8) found that the effectiveness of triple therapy for *H. pylori* declined as antral atrophy worsened. Similarly, Kalkan et al. (9) identified gastric atrophy as an independent risk factor for failure of bismuth-based quadruple therapy. Supporting these observations, studies by Shao and Xie indicated that atrophic gastritis is a significant clinical feature linked to a higher risk of antibiotic resistance in *H. pylori*-infected patients (10, 11). This link is further strengthened by Goni and colleagues, who demonstrated that primary resistance to clarithromycin in treatment-naïve patients rises proportionally with the severity of gastric atrophy (12).

Gastric mucosal atrophy typically begins in the antral pyloric gland area and spreads proximally, sequentially involving the lesser curvature of the gastric body, the cardia, the anterior and posterior walls, and finally the greater curvature (13, 14). The severity and extent of atrophy can be effectively assessed using the Kimura-Takemoto classification, a well-established endoscopic grading system (15). Based on the spread of atrophic mucosa, this system categorizes chronic atrophic gastritis into two main types: closed-type and open-type (16–18). The Kimura-Takemoto classification provides a standardized and practical endoscopic tool for evaluating gastric atrophy, showing good correlation with histological findings (19–21).

Nevertheless, high-quality clinical studies specifically examining how the endoscopic degree of atrophy affects first-line *H. pylori* eradication therapy remain relatively scarce. Consequently, this study was designed to systematically evaluate, via a single-center retrospective cohort analysis, the impact of endoscopic atrophy degree (Kimura-Takemoto open type) on the efficacy of first-line eradication therapy, and to explore whether it could serve as an independent predictor of treatment failure, thereby providing evidence-based support for individualized clinical decisions.

## 2 Materials and methods

### 2.1 Study design and participants

This was a single-center, retrospective observational cohort study. The protocol was approved by the Ethics Committee of Wuhan Fourth Hospital (Approval No: KY2026-046-01). Due to the retrospective design, individual informed consent was waived.

Using the hospital's electronic medical record system, we consecutively screened patients who presented to our hospital between November 1, 2021 and November 1, 2025, were newly diagnosed with chronic atrophic gastritis and active *H. pylori* infection, received a 14-day bismuth-containing quadruple regimen as first-line eradication therapy, and returned for a follow-up urea breath test at least 4 weeks after treatment completion to assess eradication outcome.

#### 2.1.1 Inclusion criteria

(1) Age 18–85 years;
(2) *H. pylori* infection confirmed by urea breath test;
(3) No use of other antibiotics, bismuth agents, or long-term proton pump inhibitors / H_2_ receptor antagonists within 4 weeks before treatment;
(4) Receiving first-line eradication therapy;
(5) Eradication regimen consisted of a PPI-bismuth quadruple therapy;
(6) Underwent standard high-definition white-light gastroscopy within 4 weeks before starting eradication and was diagnosed with chronic atrophic gastritis;
(7) Completed the full 14-day treatment and returned for a follow-up urea breath test at least 4 weeks after treatment completion.

The eradication regimen was a PPI-bismuth quadruple therapy: a proton pump inhibitor (esomeprazole 40 mg/day, 20 mg twice daily), a bismuth agent (bismuth aluminate granules 400 mg/day, 200 mg twice daily, containing 200 mg of bismuth element), combined with standard-dose amoxicillin (2.0 g/day, 1.0 g twice daily) and standard-dose clarithromycin (1.0 g/day, 0.5 g twice daily).

#### 2.1.2 Exclusion criteria

(1) History of gastrectomy;
(2) Pathologically confirmed gastric malignancy;
(3) Incomplete clinical records or endoscopic images;
(4) Non-compliance with follow-up;
(5) Poor medication adherence.

### 2.2 Endoscopic assessment and grouping criteria

Pre-treatment gastroscopic images of all included patients were independently and blindly reviewed by two senior endoscopists, each with over 10 years of experience in digestive endoscopy and standardized training in the Kimura-Takemoto system. Reviewers were blinded to treatment regimens, outcomes, and other clinical data.

**Figure 1:**
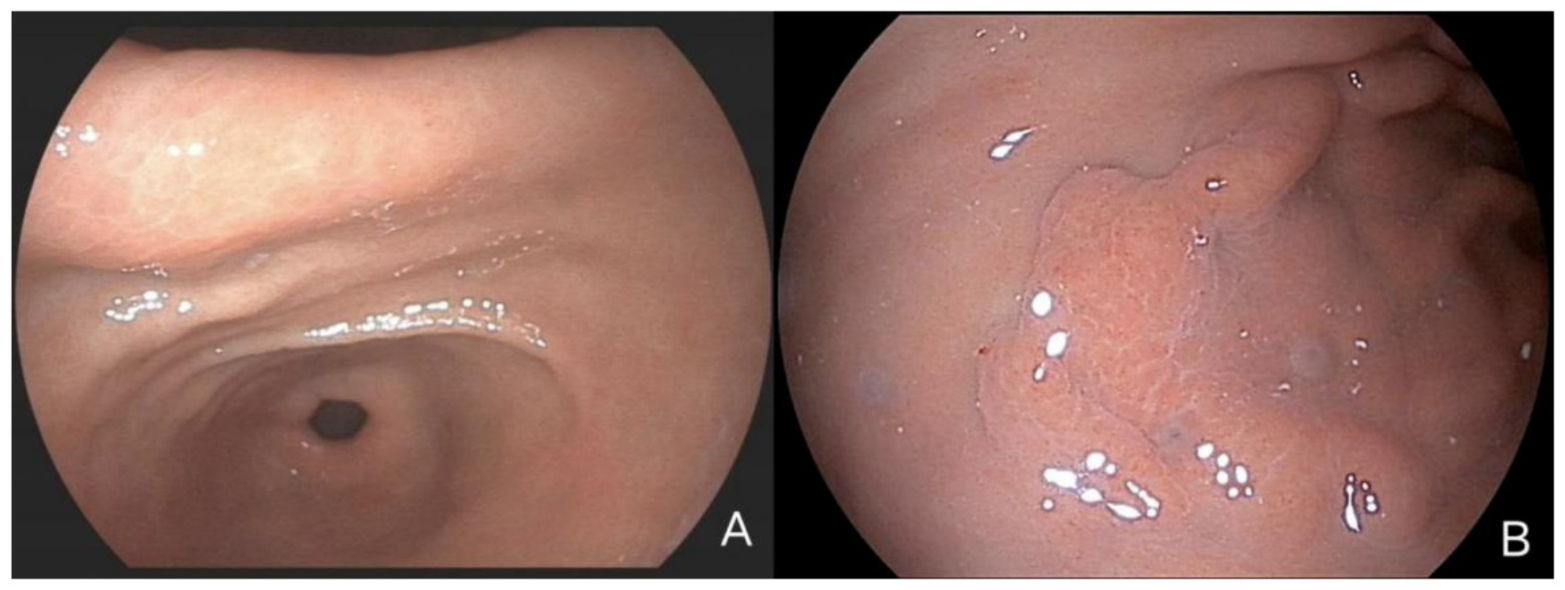
Representative endoscopic images of Kimura-Takemoto atrophy classification. **(A)** Closed-type (C-1) atrophy is confined to the antrum and the lesser curvature of the stomach. **(B)** Open-type (O-3) atrophy: atrophy extends beyond the cardia to two-thirds of the greater curvature of the gastric body.

Gastric mucosal atrophy was assessed strictly according to the Kimura-Takemoto endoscopic classification. The core exposure variable was “endoscopic atrophy type”: atrophy confined to the antrum and lesser curvature of the gastric body was defined as closed-type (C-type); atrophy extending beyond the cardia to the greater curvature of the gastric body was defined as open-type (O-type). Representative endoscopic images of closed–type (C−1) and open–type (O−3) atrophy are shown in Figure 1.

Endoscopists also recorded the presence of bile reflux, hiatal hernia, reflux esophagitis, and gastric or duodenal ulcers. Disagreements were resolved by joint re-review and discussion with reference to standard atlases. Inter-observer agreement was assessed using the Kappa statistic, which was 0.957 (95% CI: 0.898–1.012, *P* < 0.001), indicating excellent agreement.

### 2.3 Compliance assessment and study outcomes

Prescription doses were verified using outpatient pharmacy electronic dispensing records. Patient self-reported medication intake was recorded during outpatient follow-up or structured telephone interviews.

The primary outcome was *H. pylori* eradication failure. Success was defined as a negative urea breath test at least 4 weeks after completing treatment; a positive test was considered failure.

### 2.4 Data collection

The following data were prospectively collected from the hospital's electronic medical record system using a standardized case report form:

Demographics: age, sex, body mass index (BMI), education level.

Personal history: current smoking (≥1 cigarette/day on average in the 6 months before enrollment) (22, 23); current alcohol consumption (≥1 time/week in the prior 6 months) (24, 25); compliance (good if medication taken on time, poor otherwise) (26).

Comorbidities: hypertension, blood lipid levels, diabetes mellitus.

Endoscopy-related variables: atrophy type (C or O), reflux esophagitis, hiatal hernia, bile reflux, and presence of gastric or duodenal ulcers.

### 2.5 Statistical analysis

Statistical analyses were performed using SPSS version 26.0 (IBM Corp., Armonk, NY, USA). Most continuous data were normally distributed and presented as mean ± SD. Between-group comparisons used independent samples *t*-test for normally distributed data and Mann–Whitney *U*-test for non-normal data. Categorical data were expressed as frequencies (percentages) and compared using chi-square or Fisher's exact test.

To identify factors independently associated with eradication failure, we first performed univariate binary logistic regression on all candidate variables (age, sex, body weight, education, smoking, alcohol, diabetes, hypertension, serum lipids, endoscopic atrophy type, gastric ulcer, duodenal ulcer, hiatal hernia, reflux esophagitis). Variables with *P* ≤ 0.10 were entered into the initial multivariate model. Given the limited sample size (especially only 26 open-type cases), a conservative variable selection strategy was used to avoid overfitting. Results are reported as adjusted odds ratios with 95% confidence intervals. Model fit was assessed using the Hosmer–Lemeshow test (*P* ≥ 0.05 indicates good fit). All tests were two-sided, and *P* ≤ 0.05 was considered statistically significant.

## 3 Results

### 3.1 Baseline clinical characteristics of patients

**Figure 2:**
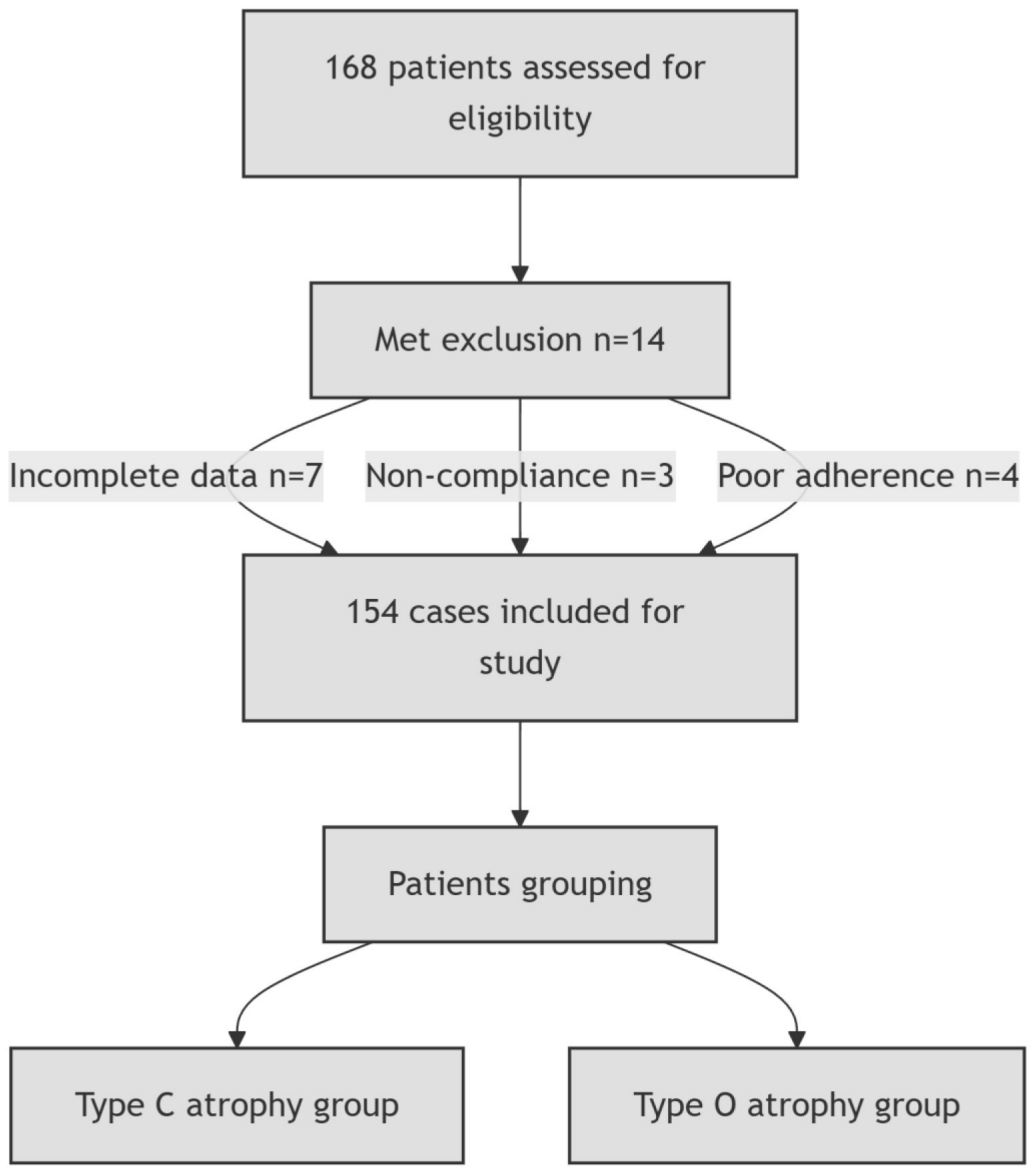
Flow chart of the study.

A total of 168 patients initially met eligibility criteria. After exclusions (seven with incomplete data, three with poor adherence, four lost to follow-up), 154 were included in the final analysis (Figure 2). Mean age was 58.12 ± 9.99 years, and 75 (48.7%) were female. No significant differences were observed between closed-type (C) and open-type (O) groups regarding demographics, comorbidities, serum lipids, or other endoscopic findings (Table 1).

**Table 1:** Baseline clinical characteristics by endoscopic atrophy type.

characteristics	Type C (*n* = 128)	Type O (*n* = 26)	*P*-value	Total (*n* = 154)
Demographics				
Age (years), mean ±SD	58.13 ± 9.83	58.65 ± 10.02	0.803	58.21 ± 9.83
Female, *n* (%)	64 (50.0)	11 (41.6)	0.474	75 (48.7)
BMI (kg/m^2^), mean ±SD	23.40 ± 3.00	23.54 ± 2.85	0.823	23.42 ± 2.96
Current smoking, *n* (%)	18 (14.1)	3 (11.5)	>0.999	21 (13.6)
Current drinking, *n* (%)	13 (10.2)	2 (7.7)	>0.999	15 (9.7)
High school diploma or higher, *n* (%)	74 (57.8)	17 (65.4)	0.474	91 (59.1)
Comorbidities				
Hypertension, *n* (%)	44 (34.4)	6 (23.1)	0.262	50 (32.5)
Diabetes, *n* (%)	19 (14.8)	4 (15.4)	>0.999	23 (14.9)
Blood lipid levels, mean ±SD				
Triglycerides (mmol/L)	1.59 ± 1.50	1.70 ± 1.23	0.751	1.60 ± 1.46
LDL cholesterol (mmol/L)	2.77 ± 0.77	2.58 ± 0.78	0.307	2.74 ± 0.77
HDL cholesterol (mmol/L)	1.27 ± 0.31	1.12 ± 0.35	0.063	1.24 ± 0.32
Total cholesterol (mmol/L)	4.68 ± 0.99	4.40 ± 1.05	0.240	4.63 ± 1.00
Endoscopic finding, *n* (%)				
Duodenal ulcer	25 (19.5)	3 (11.5)	0.415	28 (18.2)
Gastric ulcer	5 (3.9)	0 (0.0)	0.590	5 (3.2)
Reflux esophagitis	13 (10.2)	4 (15.4)	0.491	17 (11.0)
Hiatal hernia	2 (1.6)	1 (3.8)	0.428	3 (1.9)
Bile reflux	8 (6.3)	2 (7.7)	0.677	10 (6.5)

BMI, body mass index; LDL, low-density lipoprotein; HDL, high-density lipoprotein; SD, standard deviation.

### 3.2 Comparison of eradication efficacy between groups

**Figure 3:**
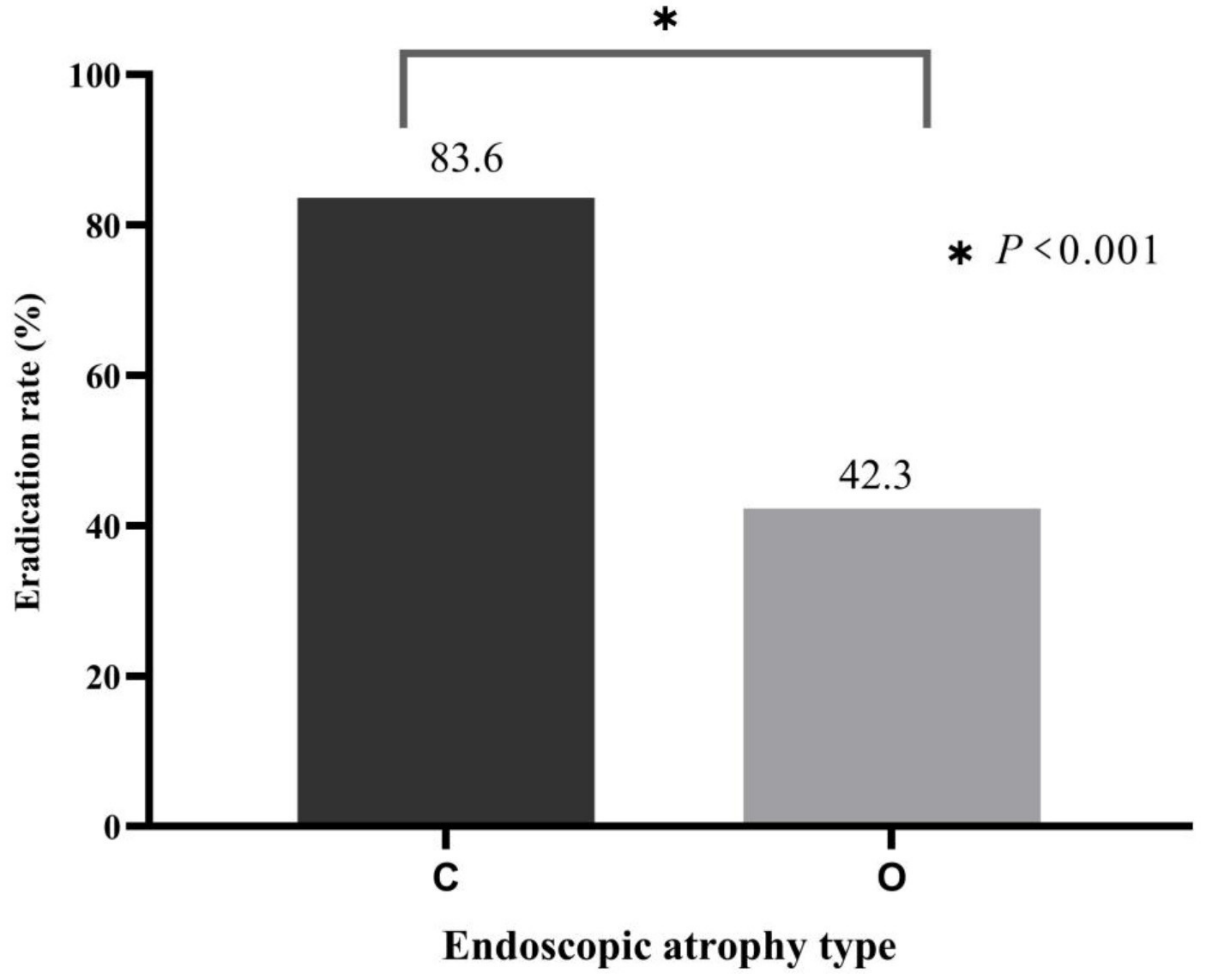
Comparison of Helicobacter pylori eradication rates between patients with closed-type (C) and open-type (O) gastric atrophy according to the Kimura-Takemoto classification. Data are presented as percentages. The brackets and asterisks indicate statistical significance (*P* < 0.001).

In the intention-to-treat analysis, the overall eradication rate was 76.6% (118/154). The closed-type group had an eradication rate of 83.6% (107/128), whereas the open-type group had a significantly lower rate of 42.3% (11/26) (χ^2^ = 20.565, *P* < 0.001; Table 2 and Figure 3).

**Table 2:** Eradication efficacy comparison between groups.

Endoscopic atrophy type	Type C (*n* = 128)	Type O (*n* = 26)	*P*-value	Total (*n* = 154)
Eradication cases	107 (83.6)	11 (42.3)	< 0.001	118 (76.6)

### 3.3 Univariate analysis of factors influencing eradication failure

Univariate logistic regression showed that BMI and endoscopic open-type atrophy were significantly associated with increased failure risk (*P* < 0.05; Table 3). Age showed a borderline association (*P* = 0.052). Sex, smoking, drinking, hypertension, diabetes, serum lipids, and presence of ulcers, hiatal hernia, or reflux esophagitis were not statistically significant (*P* > 0.10).

**Table 3:** Univariate analysis of high-risk factors associated with *Helicobacter pylori* eradication failure.

Variable	OR (95% CI)	*P*-value
Endoscopic atrophy type (Closed-type vs. Open-type)	6.948 (2.803–17.224)	< 0.001
BMI (per 1 kg/m^2^)	1.170 (1.025-1.337)	0.020
Age (per year)	0.963 (0.927–1.000)	0.052
Reflux esophagitis (absent vs. present)	1.945 (0.665–5.694)	0.225
Female (no vs. yes)	0.926 (0.438–1.954)	0.839
Current smoking (no vs. yes)	0.743 (0.233–2.368)	0.615
Current drinking (no vs. yes)	0.803 (0.214–3.018)	0.745
High school diploma (no vs. yes)	1.301 (0.601–2.815)	0.504
Hypertension (no vs. yes)	0.891 (0.398–1.996)	0.780
Diabetes (no vs. yes)	1.188 (0.430–3.282)	0.739
Triglycerides (per 1 mmol/L)	1.074 (0.838–1.375)	0.572
LDL cholesterol (per 1 mmol/L)	0.915 (0.549–1.527)	0.735
HDL cholesterol (per 1 mmol/L)	0.543 (0.154–1.920)	0.344
Total cholesterol (per 1 mmol/L)	0.846 (0.565–1.266)	0.415
Duodenal ulcer (no vs. yes)	0.873 (0.324–2.352)	0.788
Gastric ulcer (no vs. yes)	2.255 (0.362–14.052)	0.384
Hiatal hernia (absent vs. present)	1.657 (0.146-18.824)	0.684
Bile reflux (absent vs. present)	0.809 (0.164–3.992)	0.794

OR, odds ratio; CI, confidence interval; BMI, body mass index; LDL, low-density lipoprotein; HDL, high-density lipoprotein.

### 3.4 Multivariate analysis of factors associated with eradication failure

**Figure 4:**
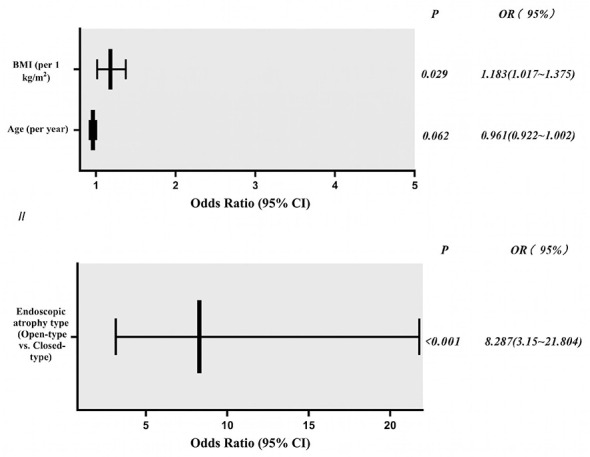
Forest plot of independent associated factors for *H. pylori* eradication failure. For clearer presentation, the X-axis scale has been adjusted. The upper panel shows the detailed distribution of BMI and age (0–5), and the lower panel shows the full distribution of atrophy type (0–22).

Only the three variables with *P* < 0.10 (BMI, age, and endoscopic atrophy type) were entered into the multivariate model. After adjusting for age and BMI, open-type atrophy remained the most significant independent factor (OR = 8.287, 95% CI: 3.150–21.804, *P* < 0.001; Table 4 and Figure 4). BMI was also independently associated (each 1 kg/m^2^ increase linked to an 18.3% higher failure risk, OR = 1.183, 95% CI: 1.017–1.375, *P* = 0.029). Age was not significant in the multivariate model (*P* = 0.062). The Hosmer–Lemeshow test indicated good model fit (χ^2^ = 11.623, *P* = 0.169).

**Table 4:** Multivariate logistic regression analysis of independent associated factors for *H. pylori* eradication failure.

Variable	OR (95% CI)	*P*-value
Endoscopic atrophy type (Open-type vs. Closed-type)	8.287 (3.150–21.804)	< 0.001
BMI (per 1 kg/m^2^)	1.183 (1.017–1.375)	0.029
Age (per year)	0.961 (0.922–1.002)	0.062

OR, adjusted odds ratio; CI, confidence interval; BMI, body mass index.

Model fit: Hosmer–Lemeshow test, χ^2^ = 11.623, *P* = 0.169.

Variables with *P* < 0.10 in univariate analysis were entered into the multivariate model (endoscopic atrophy type, BMI, age).

**Figure 5:**
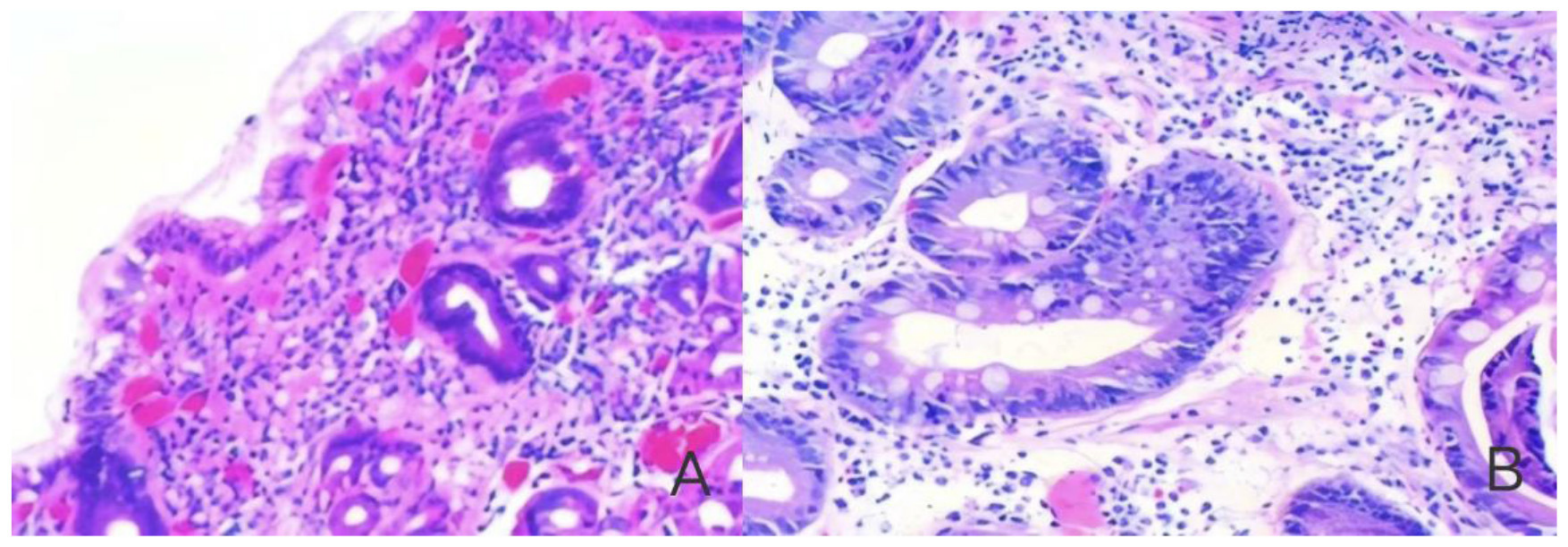
Histopathological findings of gastric mucosal atrophy (HE staining, × 200). **(A)** Mild atrophy: mild reduction of gastric glands and sparse chronic inflammatory cell infiltration. **(B)** Severe atrophy (corresponding to open-type atrophy): marked loss of gastric glands with extensive intestinal metaplasia.

To further support the endoscopic assessment, we compared histopathological findings between mild and severe atrophy. As shown in Figure 5, severe atrophy (corresponding to open–type endoscopic findings) was characterized by marked loss of gastric glands with extensive intestinal metaplasia.

## 4 Discussion

**Figure 6:**
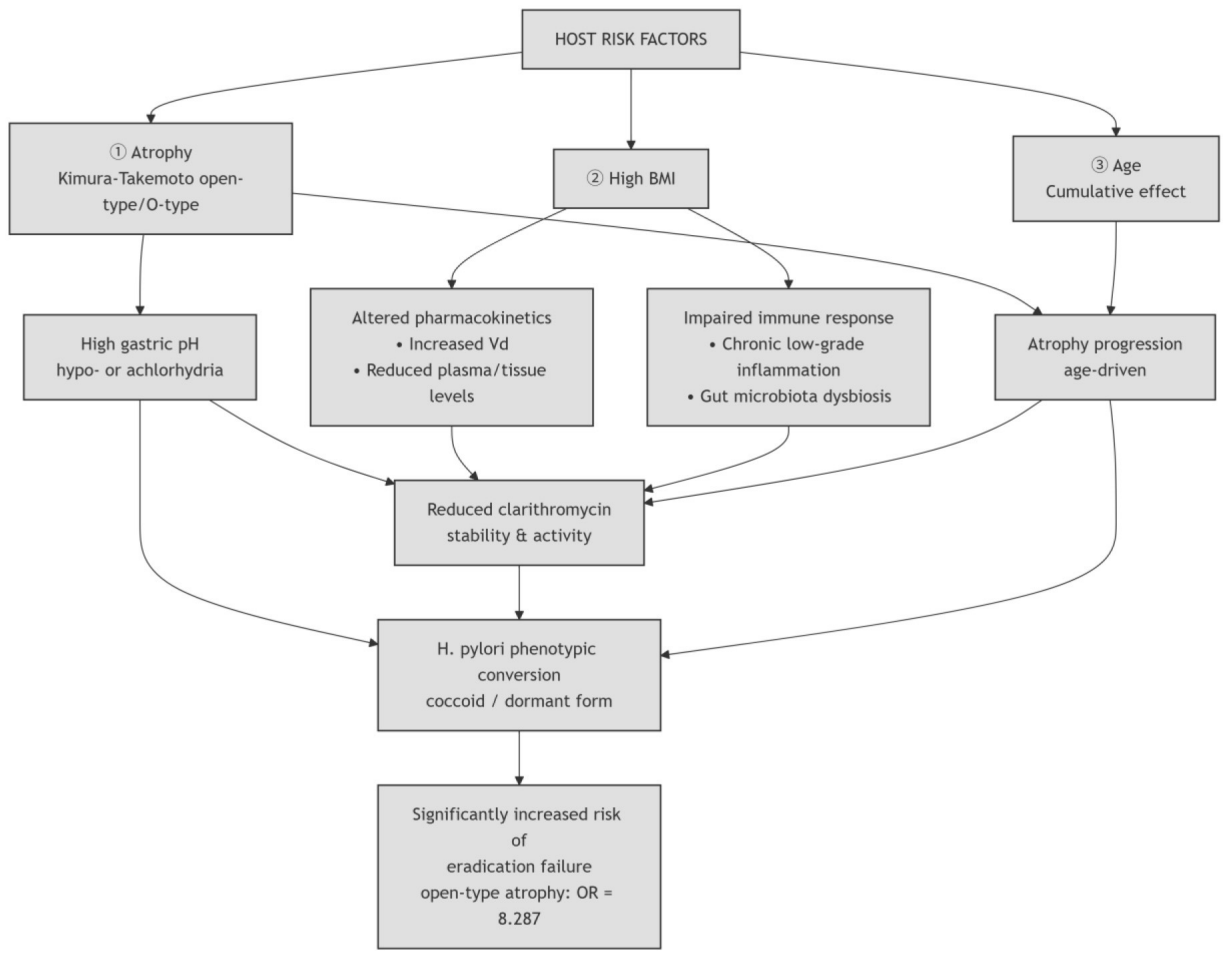
Conceptual diagram of risk factors and their cumulative impact on *H. pylori* first-line eradication failure. This figure integrates the three main host risk factors identified in this study (open-type atrophy, high BMI, and age) and their mechanistic interactions. Open-type atrophy leads to a high intragastric pH environment, reducing the stability and antibacterial activity of clarithromycin and promoting the phenotypic conversion of *H. pylori* to a dormant coccoid form. High BMI further impairs treatment efficacy by altering antibiotic pharmacokinetics (increased volume of distribution, reduced plasma/tissue concentrations) and impairing immune response. Age serves as an important confounding factor, indirectly influencing eradication outcomes by driving the cumulative progression of atrophy. The additive effects of these factors ultimately lead to a significantly increased risk of eradication failure (open-type atrophy: OR = 8.287, 95% CI: 3.150 –21.804, *P* < 0.001).

Our findings indicate that, in patients treated with first-line regimens, the endoscopic finding of open-type gastric atrophy (Kimura-Takemoto type O) is associated with an independently elevated risk of eradication failure - roughly 8.3 times higher. The conceptual diagram in Figure 6 visually summarizes the mechanisms linking the identified risk factors to treatment failure.

The key finding-that open-type atrophy significantly reduces the first-line eradication success of a clarithromycin-containing bismuth-based quadruple regimen-has a solid pathophysiological basis. Three interrelated mechanisms likely contribute: pharmacokinetic alterations, selection of resistant strains, and bacterial phenotypic adaptation, all linked to the severe hypoxic environment caused by open-type atrophy.

First, open-type atrophy leads to extensive loss of parietal cells in the gastric body, resulting in severely impaired acid secretion or even achlorhydria (27, 28). This hypochlorhydric environment negatively impacts clarithromycin, a macrolide antibiotic whose solubility and stability are pH-dependent. At higher intragastric pH, clarithromycin solubility decreases, and the drug becomes more susceptible to degradation, reducing oral absorption and mucosal concentration, thereby diminishing antibacterial activity (29, 30). In our study, the standard clarithromycin dose (1.0 g/day) was used, and its pharmacokinetic disadvantage was amplified in a hypo-acid environment, explaining the stark difference in eradication rates between open-type (42.3%) and closed-type (83.6%) groups. Notably, amoxicillin (a β-lactam) is relatively pH-stable, but its efficacy depends on actively proliferating bacteria (31–34). The hypo-acid environment can induce *H. pylori* to enter a dormant phase, indirectly reducing amoxicillin's effectiveness (35, 36).

Second, progressive atrophy is not merely a morphological change but also a “breeding ground” for drug-resistant strains (10, 11). Goni et al. (12) found that primary clarithromycin resistance rates rise with increasing atrophy severity in treatment-naïve patients. Although our open-type patients were receiving first-line therapy, chronic exposure to an atrophic microenvironment may have predisposed them to harbor a higher proportion of clarithromycin-resistant strains. This phenomenon may involve *H. pylori* virulence factors: CagA-positive strains not only induce atrophy but also promote biofilm formation, a key resistance mechanism against multiple antibiotics including clarithromycin (37, 38). Consequently, open-type patients may naturally carry resistant strains and simultaneously experience reduced clarithromycin efficacy due to elevated intragastric pH, rendering standard quadruple therapy largely ineffective.

Third, the hypo-acid, atrophic environment can induce phenotypic conversion of *H. pylori* from spiral to coccoid dormant forms (39, 40). Coccoid forms exhibit extremely low metabolic activity and are insensitive to both β-lactams (e.g., amoxicillin) and macrolides (e.g., clarithromycin), acting as a potential reservoir for post-eradication recurrence (35, 36).

Beyond endoscopic open-type atrophy, we found that higher BMI was independently associated with first-line eradication failure (OR = 1.183 per 1 kg/m^2^, 95% CI: 1.017-1.375, *P* = 0.029). This aligns with clinical evidence showing progressively lower eradication rates with increasing obesity severity (41). Mechanistically, obesity affects antibiotic pharmacokinetics: the expanded volume of distribution for lipophilic drugs like clarithromycin reduces plasma and tissue concentrations with standard dosing (42–44). Additionally, obesity-related chronic low-grade inflammation and gut microbiota dysbiosis may impair immune responses (45). Although we did not measure drug levels or inflammatory markers, these mechanisms provide a plausible explanation for our findings. For patients with elevated BMI, especially severe obesity, weight-adjusted dosing or alternative regimens may be considered.

Regarding age, univariate analysis showed a borderline association with eradication failure (*P* = 0.052), but this did not persist in multivariate analysis (*P* = 0.062). This finding merits discussion. Given that atrophic mucosal changes develop cumulatively over a lifespan due to long-term persistence of *H. pylori* infection (46, 47), age is likely a powerful confounding factor influencing chronic tissue remodeling in the stomach. The loss of statistical significance in multivariate analysis may be explained by collinearity between age and atrophy type: older patients are more likely to have open-type atrophy, and once atrophy grade is accounted for, the independent effect of age may be attenuated. Alternatively, the limited sample size (especially only 26 open-type cases) may have underpowered the detection of a modest independent effect of age. Future larger-scale studies are needed to disentangle the independent contributions of age and atrophy severity.

In summary, multiple mechanisms-from clarithromycin's pharmacokinetic impairment due to elevated gastric pH, to the selective advantage of resistant strains in an atrophic microenvironment, to bacterial coccoid transformation—collectively explain the poor response of open-type atrophy patients to clarithromycin-containing quadruple therapy. Our findings align with those of Kalkan et al. (“gastric atrophy as an independent risk factor for eradication failure”) and Goni et al. (“atrophy severity correlates with primary clarithromycin resistance”), and for the first time clearly demonstrate at the clinical level the strong negative impact of endoscopic open-type atrophy on first-line eradication. Therefore, for patients with endoscopic open-type atrophy, continued empirical use of a clarithromycin-containing quadruple regimen may not be optimal; alternative regimens (e.g., avoiding clarithromycin) or susceptibility-guided individualized therapy should be considered.

### 4.1 Clinical implications

Pre-treatment endoscopy value: our findings support performing diagnostic gastroscopy before first-line *H. pylori* eradication when feasible. Endoscopic assessment of atrophy extent (closed vs. open) can serve as a simple, intuitive tool to predict treatment outcomes and guide clinical decisions.

Individualized therapy for high-risk patients: for patients with open-type atrophy (type O), the clarithromycin-containing bismuth quadruple regimen achieved only a 42.3% eradication rate, far below the 83.6% in closed-type patients. We explicitly recommend not using this regimen empirically as first-line therapy for Kimura-Takemoto open-type atrophy. Alternative strategies include: (1) clarithromycin–free regimens (e.g., tetracycline–based, metronidazole–based, levofloxacin–based, or high–dose amoxicillin with PPI/vonoprazan); (2) if possible, perform gastric biopsy and antibiotic susceptibility testing before treatment; (3) for patients with high BMI, consider dose adjustment or prolonged treatment. These strategies may improve eradication success in this population.

### 4.2 Limitations

Several limitations should be considered. First, as a single-center retrospective study, selection and information bias are possible; loss to follow-up may have influenced results. Second, antibiotic susceptibility testing was not performed, so confounding due to uneven distribution of primary resistant strains cannot be fully excluded. Third, although endoscopic atrophy assessment was performed by two experienced endoscopists with blinding and high inter-observer agreement (kappa = 0.957), the Kimura-Takemoto classification remains a visual scale with inherent subjectivity and does not provide 100% specificity. More importantly, we did not perform parallel histological evaluation of biopsies (e.g., using the OLGA/OLGIM systems) in this retrospective cohort, as standardized biopsy protocols were not available for all patients. Endoscopic appearance of “atrophy” may not perfectly mirror the true morphological loss of parietal cells and gastric glands, as illustrated by the histopathological comparison in Figure 5. Therefore, future prospective studies incorporating histopathological confirmation are needed to validate the accuracy of endoscopic atrophy grading in predicting treatment outcomes. Fourth, the sample size was relatively small, especially the open-type group (*n* = 26), which may be underpowered to detect modest independent effects (e.g., age) and may affect the stability of subgroup analyses. Fifth, in multivariate analysis, we only included three variables with univariate *P* < 0.10 (atrophy type, BMI, age). Although this strategy aimed to control overfitting, it may have omitted clinically important variables with slightly higher *P* values (e.g., HDL cholesterol had a baseline *P* = 0.063 but univariate regression *P* = 0.344). Thus, variable selection bias is possible. Larger prospective studies are needed to validate our findings and more comprehensively assess confounders.

## 5 Conclusion

Both a high BMI and the presence of open-type atrophy (Kimura-Takemoto type O) independently predict poorer outcomes following first-line *H. pylori* eradication therapy. Based on these findings, we recommend that clarithromycin-containing bismuth-based quadruple regimens be used with caution as first-line therapy for patients with Kimura-Takemoto open-type atrophy. Alternative clarithromycin-free regimens (e.g., tetracycline-, metronidazole-, or levofloxacin-based therapies, or high-dose amoxicillin with PPI/vonoprazan) or susceptibility-guided individualized therapy are preferred for such patients.

## Data Availability

The raw data supporting the conclusions of this article will be made available by the authors, without undue reservation.
